# The Structure of the Oligomerization Domain of Lsr2 from *Mycobacterium tuberculosis* Reveals a Mechanism for Chromosome Organization and Protection

**DOI:** 10.1371/journal.pone.0038542

**Published:** 2012-06-13

**Authors:** Emma L. Summers, Kathrin Meindl, Isabel Usón, Alok K. Mitra, Mazdak Radjainia, Roberto Colangeli, David Alland, Vickery L. Arcus

**Affiliations:** 1 Department of Biological Sciences, University of Waikato, Hamilton, New Zealand; 2 Instituto de Biología Molecular de Barcelona, Barcelona Science Park, Barcelona, Spain; 3 Institucio Catalana de Recerca i Estudis Avançats at Instituto de Biología Molecular de Barcelona, Barcelona Science Park, Barcelona, Spain; 4 School of Biological Sciences, University of Auckland, Auckland, New Zealand; 5 Division of Infectious Disease and the Center for Emerging Pathogens, Department of Medicine, New Jersey Medical School, University of Medicine and Dentistry of New Jersey, Newark, New Jersey, United States of America; Saint Louis University, United States of America

## Abstract

Lsr2 is a small DNA-binding protein present in mycobacteria and related actinobacteria that regulates gene expression and influences the organization of bacterial chromatin. Lsr2 is a dimer that binds to AT-rich regions of chromosomal DNA and physically protects DNA from damage by reactive oxygen intermediates (ROI). A recent structure of the C-terminal DNA-binding domain of Lsr2 provides a rationale for its interaction with the minor groove of DNA, its preference for AT-rich tracts, and its similarity to other bacterial nucleoid-associated DNA-binding domains. In contrast, the details of Lsr2 dimerization (and oligomerization) via its N-terminal domain, and the mechanism of Lsr2-mediated chromosomal cross-linking and protection is unknown. We have solved the structure of the N-terminal domain of Lsr2 (N-Lsr2) at 1.73 Å resolution using crystallographic *ab initio* approaches. The structure shows an intimate dimer of two ß–ß–a motifs with no close homologues in the structural databases. The organization of individual N-Lsr2 dimers in the crystal also reveals a mechanism for oligomerization. Proteolytic removal of three N-terminal residues from Lsr2 results in the formation of an anti-parallel β-sheet between neighboring molecules and the formation of linear chains of N-Lsr2. Oligomerization can be artificially induced using low concentrations of trypsin and the arrangement of N-Lsr2 into long chains is observed in both monoclinic and hexagonal crystallographic space groups. In solution, oligomerization of N-Lsr2 is also observed following treatment with trypsin. A change in chromosomal topology after the addition of trypsin to full-length Lsr2-DNA complexes and protection of DNA towards DNAse digestion can be observed using electron microscopy and electrophoresis. These results suggest a mechanism for oligomerization of Lsr2 via protease-activation leading to chromosome compaction and protection, and concomitant down-regulation of large numbers of genes. This mechanism is likely to be relevant under conditions of stress where cellular proteases are known to be upregulated.

## Introduction

Bacterial nucleoid-associated proteins (NAPs) play important roles in chromosome organization and gene regulation [1]–[4]. Histone nucleoid structuring protein (H-NS) is an abundant non-specific DNA-bridging NAP in the *Enterobacteriaceae* and is thought to have a role in global gene expression [1]. The NAP histone-like protein, HU (heat unstable), also binds DNA non-specifically and introduces bends in the DNA, and this effect is enhanced in the absence of H-NS [5]. In mycobacteria, NAPs such as the histone like protein, H-NS and Lsr2 have been identified and studied in some detail from *Mycobacterium tuberculosis* and *Mycobacterium smegmatis* [6]–[8].

Lsr2 from *M. tuberculosis* is a small, basic protein (M_r_ = 12.1 kDa, pI = 10) that is highly conserved in mycobacteria and related actinobacteria [9]. Lsr2 was first identified as an immunodominant T-cell antigen of *M. leprae* [10]–[12] and Lsr2 orthologues are present in all sequenced mycobacterial genomes. Lsr2 orthologues have also been identified in a number of mycobacteriophage genomes [13], [14].

Lsr2 is thought to be regulated by iron and microarray studies have shown that Lsr2 is up-regulated at high temperatures and upon nutrient starvation [15], [16]. A range of biological functions have been described for Lsr2. It down-regulates genes involved in cell wall synthesis and metabolic functions in *M. smegmatis* [17]. Knock-out mutants of Lsr2 in *M. smegmatis* show altered colony morphology and reduced biofilm formation [18], [19]. Lsr2 cannot be deleted from the *M. tuberculosis* genome suggesting that it is essential in this important human pathogen [20].

Lsr2 is a DNA-binding protein that likely influences the organization of bacterial chromatin and gene regulation by binding to AT-rich segments of DNA [7]. Lsr2 is believed to have roles beyond gene expression. It has been shown to physically protect DNA by binding and shielding it from degradation by reactive oxygen species [20]. Lsr2 is thought to be functionally related to the Gram-negative bacterial histone-like proteins [21] and can complement H-NS knock-out mutants in *Escherichia coli.* Reverse complementation is also possible by H-NS in Lsr2 knock-out mutants of *M. smegmatis* [21]. The construction of H-NS/Lsr2 chimeras that rescue H-NS deletion mutants in *E. coli* reinforce this functional relationship [21]. Recent NMR structures of the C-terminal DNA-binding domains of Lsr2 and H-NS revealed topological similarities between these domains and a “Q/RGR” amino acid motif responsible for DNA minor-groove binding and conferring specificity for AT-rich DNA sequences [22], [23]. These structures were used to propose a model for DNA-binding which suggested that Lsr2 (as well as H-NS) binds in two orientations by grabbing either edge of the DNA minor groove like a clamp [22], [23]. It should be noted however, that secondary structure predictions suggest significant differences between the N-terminal domains of H-NS and Lsr2. Intriguingly, the recent structure of the N-terminal domain of H-NS showed a super-helical arrangement and provided a model of chromosome organization by H-NS in enterobacteria [24].

Here, we report the structure of the N-terminal dimerization domain of Lsr2 solved by X-ray crystallography at 1.73 Å resolution using *ab initio* methods. The structure shows tightly packed dimers that link via the association of N-terminal ß-strands to form long, linear chains. The formation of these linear chains (oligomerization) requires the loss of three N-terminal residues and presents a mechanism for oligomerization of Lsr2 via proteolytic activation. Both the domain structure and the mode of oligomerization for Lsr2 are entirely different from that of H-NS. We show that the self-association of full-length Lsr2 can be triggered by using low concentrations of trypsin. In addition, we observe by electron microscopy that proteolysis drives compaction of DNA-associated Lsr2 into both disordered “knots” and more regular DNA-Lsr2 fibrils. It is known that various intracellular proteases are upregulated in mycobacteria in response to stress factors including temperature and antibiotic challenge. Thus, the structure of the Lsr2 N-terminal dimer and our observation of the self-associative propensity of this dimer points towards a mechanism for Lsr2-mediated chromosome compaction and gene regulation in response to external stress.

## Results

### Crystallization and Structure Solution using Ab Initio Methods

Several constructs of the Lsr2 N-terminal domain (N-Lsr2) were made that incorporated increasing numbers of residues from the linker region between the N-terminal and C-terminal domains (Table 1). Crystals grew from one of these constructs (Nterm2) after a few days but this was dependent on “maturation” of the protein sample (by some unknown process) over a long period of time. At first, we did not fully understand why crystals could only grow from relatively old protein samples. In retrospect, this “maturation” process amounted to degradation of Lsr2 at Lys-4 to generate a truncation product with Lys-4 as the new N-terminal residue. Highly redundant crystallographic data were collected from these crystals to 1.73 Å resolution. A small unit cell and the presence of a self-rotation peak in the Patterson map suggested a dimer in the asymmetric unit with low solvent content (39%) in the unit cell. Given the high quality data and the predicted presence of alpha helices, the data were suitable for recently developed *ab initio* methods for structure solution [25].

**Table 1:** The C-terminal boundaries of Lsr2 N-terminal domain constructs.

Construct Name	Amino Acid Sequence (from residue 55 onwards)	No. Amino Acids
Nterm	55 – G R R V G	59
Nterm+	55 – G R R V G G R R R G R S G S	68
Nterm1	55 – G R	56
Nterm2	55 – G R R V G G R	61
Nterm3	55 – G R R V G G R R R G	64
Nterm4	55 – G R R V G G R R R G R S G S G R G	71
Nterm5	55 – G R R V G G R R R G R S G S G R G R G A	74

The structure in the monoclinic space group *P*2_1_ was solved at 1.8 Å using the latest version of the ARCIMBOLDO program [26]. A 14-residue long model polyalanine alpha-helix was used as the search fragment to arrive at the *ab initio* solution. Location of the first fragment in the structure with PHASER [27] rendered 26 solutions, characterized by very similar figures of merit. None of the 10 selected for the expressway was effective in expanding to the full structure. Neither did the expansion of the 10 prioritized solutions of two fragments with SHELXE [28] among the 95 partial solutions located with PHASER [27], lead to a solution. Still, a difference of 4% in the correlation coefficient (CC) of a few solutions, compared to the CC values for the rest led to their identification as promising candidates. Iterations of pdb optimization (eliminating residues from the trace whenever this lead to an increase in the CC for the fragment), and expansion with SHELXE (using 30 cycles of density modification, no sharpening, extrapolating missing reflections to 1 Å and 3 cycles of autotracing) afforded main chain traces with CC values over 30% in 1 of the 10 selected cases. This initial structure represented a solution from which the remainder of the model could be built.

A second structure generated from crystals in the hexagonal space group *P*3_1_21 was solved at 2.04 Å resolution. These crystals contained protein truncated at Lys-4 as a result of limited proteolysis using trypsin. These crystals had a higher solvent content (70%) and one N-Lsr2 dimer in the asymmetric unit.

### The Structure of the N-terminal Domain of Lsr2

Each of the two crystal structures of the *M. tuberculosis* Lsr2 (Rv3597c) N-terminal dimerization domain contained two copies of the protein in the asymmetric unit forming an intimate dimer that comprises residues Lys-4 toVal-58 in chain A and Lys-4 to Gly-59 in chain B (the RMSD between chains A and B is 0.23 Å). Two copies of the protein in the asymmetric unit in space group *P*2_1_ results in a Matthew’s coefficient of 2.0 Å^3^.Da^−1^ and a solvent content of 39.8% whereas the solvent content in the *P*3_1_21 crystals was relatively high at 70.0% with a Matthew’s coefficient of 4.1 Å^3^.Da^−1^. No interpretable electron density was observed for the N-terminal fusion tag, the N-terminal residues Met-1, Ala-2, Lys-3 or for the C-terminal residues Gly-60 and Arg-61. We subsequently learned that these absent residues at the N-terminus had been cleaved by contaminating proteases in the case of the *P*2_1_ crystals. We mimicked this process by the deliberate addition of trace amounts of trypsin in the case of the *P*3_1_21 crystals. The final models have crystallographic *R*-factors of 0.160 and 0.251 and *R_free_* factors of 0.185 and 0.287, for *P*2_1_ and *P*3_1_21 crystal forms respectively and display excellent stereochemistry. For the *P*2_1_ and *P*3_1_21 crystal forms, 99% and 100% of residues lie in the favoured regions of the Ramachandran plot respectively, and there are no residues in disallowed regions. The Lsr2 structures from the two different space groups are very similar with an RMSD of 0.54 Å for all protein atoms (comparing dimers in each space group). A summary of the data collection and refinement statistics is given in Table 2. The differences in R-factors between the two structures may reflect different levels of precision in the two models (as evidenced by Cruickshanks DPI for each model). However, these global statistics may also be influenced by the large differences in solvent content and different crystal symmetries between the two structures.

**Table 2:** Crystallographic data collection and refinement statistics.

	Nterm2	Nterm2 + Trypsin
*Data Collection*		
Wavelength (Å)	1.5417	0.9786
Space Group	P1 2_1_ 1	P3_1_ 2 1
Unit Cell Dimensions (Å)	32.51, 27.03, 56.83	57.39, 57.39, 105.31
Unit Cell Angles (°)	90.0, 94.3, 90.0	90.0, 90.0, 120.0
Resolution (Å)^a^	32.42–1.73 (1.82–1.73)	52.65–2.04 (2.15–2.04)
R-merge	0.092 (0.308)	0.096 (0.562)
Completeness (%)	99.4 (96.3)	100.0 (100.0)
Redundancy	9.77 (9.18)	20.43 (20.45)
No. of Observations	102957 (13617)	272612 (38858)
No. of Unique Reflections	10538 (1483)	13341 (1900)
Mean I/σ(I)	17.1 (6.9)	22.6 (6.2)
*Refinement*		
R-factor	0.160 (0.195)	0.251 (0.283)
R-free	0.185 (0.253)	0.287 (0.337)
Cruickshanks DPI (Å)	0.114	0.179
Protein Atoms	834	838
Solvent Atoms	206	135
Average B-value (Å^2^)	13.6	36.7
RMSD		
Bond Angles (°)	0.914	0.997
Bond Lengths (Å)	0.006	0.008

a Numbers in parentheses correspond to the highest resolution shell.

**Figure 1:**
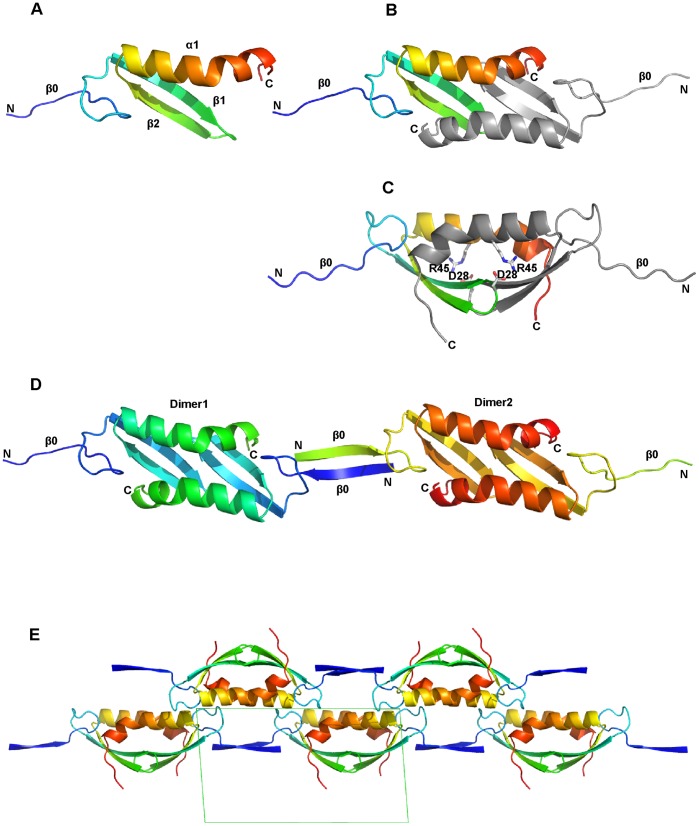
Cartoon diagrams of the Lsr2 N-terminal domain in the *P*2_1_ crystal structure. (A) A depiction of the monomer: the α-helix, β-strands and chain termini are labeled and the chain is colored blue-red (N-terminus to C-terminus); (B) The dimer as seen in the crystal structure, one chain is colored and the other is grey; (C) A view orthogonal to that of B showing residues critical for dimerization; (D) Lsr2 N-terminal domain oligmerization as generated by crystallographic symmetry. The N-terminus of one dimer donates one strand forming an anti-parallel β-sheet. The second strand is presented by a neighboring dimer. The β-sheet linking two dimers is shown as β0; (E) Crystallographic symmetry (in space group *P*2_1_) showing the unit cell (in green) projected perpendicular to the b-axis. The two-fold screw axis generates alternating Lsr2 chains that lie back-to-back along the horizontal (in this view). For all figures protein depictions were drawn with PYMOL.

**Figure 2:**
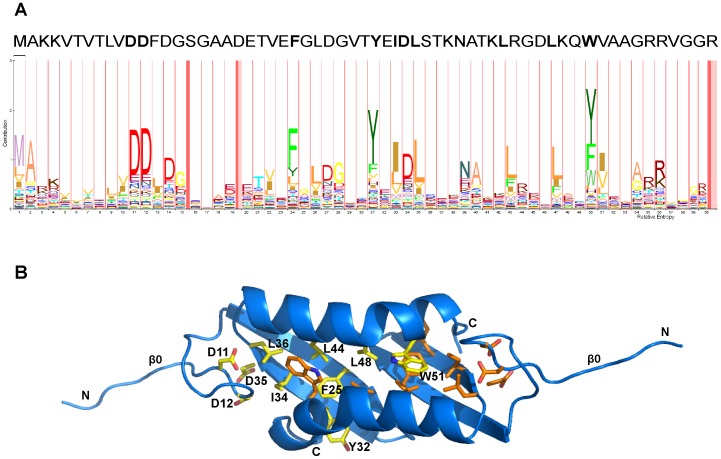
Conserved amino acids for the N-terminal domain of Lsr2. (A) Relative conservation of amino acids at different positions from a multiple sequence alignment are depicted using HMM Logo (http://pfam.sanger.ac.uk/family/PF11774.2). The height of the letters represents the relative entropy and the width represents the relative contribution of the position to the overall protein family. The pink bars represent regions of insertion in the alignment. Amino acid residue colours reflect their biological propertes (red  =  charged; blues  =  polar, uncharged; yellows  =  aliphatic; greens  =  aromatic). The amino acid sequence for Lsr2 from *M. tuberculosis* is shown across the top of the HMM logo for comparison and residues that are well-conserved are in bold. (B) Cartoon diagram of Lsr2 N-terminal dimerization domain showing conserved residues. The structure is for the *P*2_1_ crystal form. Conserved residues for each chain are shown in yellow and orange respectively. Yellow residues from chain A are labeled. The chain termini are also labeled.

The Lsr2 dimerization domain forms a compact homodimeric α/β structure (Figure 1A-C). The monomer displays an extended N-terminus followed by two strands of an anti-parallel β-sheet and an α-helix with a significant kink. The N-termini of the β-sheet and the α-helix are adjacent and they pack with their long axes at an angle of approximately 45° (Figure 1A). The dimer forms a 4-stranded anti-parallel β-sheet such that the helices are arranged in an antiparallel fashion and are on the same side of the β-sheet (Figure 1B). The kinked C-terminus of the helix allows it to pack against the ß-strands of the neighboring molecule in the dimer. Residues that have previously been implicated in dimerization – Asp-28 and Arg-45 [7] – anchor the anti-parallel ß-sheet of one chain to the alpha-helix of the other chain via a salt bridge (Figure 1C). The dimer interface buries 1548 Å^2^, corresponding to 31% of the total surface area of each monomer. Ten residues are well conserved across the N-terminal domain of the Lsr2 protein family (Asp-11, Asp-12, Phe-25, Tyr-32, Ile-34, Asp-35, Leu-36, Leu-44, Leu-48 and Trp-51, Figure 2). These cluster in the core of the dimer and provide a large number of tertiary hydrophobic and H-bonding interactions that connect the ß-sheet, alpha-helix and a large N-terminal loop (Figure 2B).

### Oligomerization of Lsr2 Dimerization Domain

**Figure 3:**
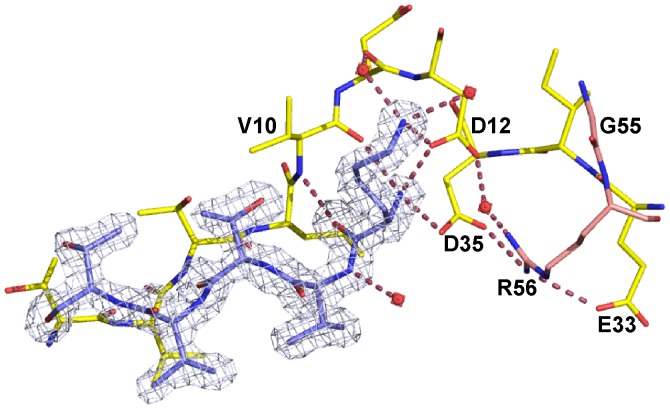
The oligomeric interface. The interaction between Lys-4 of one monomer (in blue) and the neighboring N-Lsr2 dimer (in yellow and pink). This interaction is present in both crystal forms. Polar interactions are labelled with pink dotted lines and selected residues are labeled. An electron density map contoured at 1σ is shown as blue mesh.

The monoclinic crystal structure reveals a linear chain of dimers formed by crystallographic symmetry (Figures 1D & E). The N-terminal end of each dimer forms a single ß-strand (ß0) that is then paired with the N-terminal strand of a neighboring dimer (Figures 1D & E) to form the linear oligomer chain. The oligomer is not only stabilized by interstrand H-bonds, but also by a network of polar interactions between Lys-4 (the new N-terminus after truncation) and its oligomeric neighbor (Figure 3). Both positively charged primary amino groups of Lys-4 (from one dimer) form a suite of polar interactions with carbonyl groups, acidic groups and water molecules (from the neighboring dimer). This structural arrangement could not form if the N-terminal residues Met-1, Ala-2 and Lys-3 were present. Firstly, steric constraints preclude an amino acid at the Lys-3 position and secondly, the three polar interactions between the Lys-4 backbone amide and Val-10, Asp-12 and Asp-35 could not form if a peptide bond were present (Figure 3). Thus, the structure led us to the hypothesis that post-translational proteolytic processing of Lsr2 (removing Met-1 to Lys-3) is required for oligomerization leading to the formation of long N-Lsr2 polymer chains.

We then artificially induced this proteolytic processing by adding low concentrations of trypsin to purified N-Lsr2 before crystallization. This facilitated crystallization in a different space group, *P*3_1_21. The dimerization and the linear oligomerization of N-Lsr2 is identical in this hexagonal space group when compared to the structure in the *P*2_1_ crystals. The difference between the two space groups is the arrangement of the linear chains of N-Lsr2. The linear oligomer strands lie side-by-side in the monoclinic (*P*2_1_) crystals whereas the hexagonal crystals (*P*3_1_21) are made up of stacked layers of linear strands that are rotated by 120 degrees (Figure 1E). This provides further evidence that the tendency to form linear chains of dimers by processing of the N-terminus is intrinsic and not a crystallographic artifact.

We checked the N-terminal proteolytic processing by using electrospray mass spectrometry to obtain accurate masses for proteins from both the monoclinic crystals (N-Lsr2 processed by contaminating proteases) and the trypsinized hexagonal crystals (N-Lsr2 processed by a low concentration of trypsin). These masses were 6198.9 Da for the protein from monoclinic crystals and 6200.1 Da for protein from the hexagonal crystals. These figures compare closely to the calculated molecular weight of 6199.8 Da for Lys4-Arg58 N-Lsr2.

**Figure 4:**
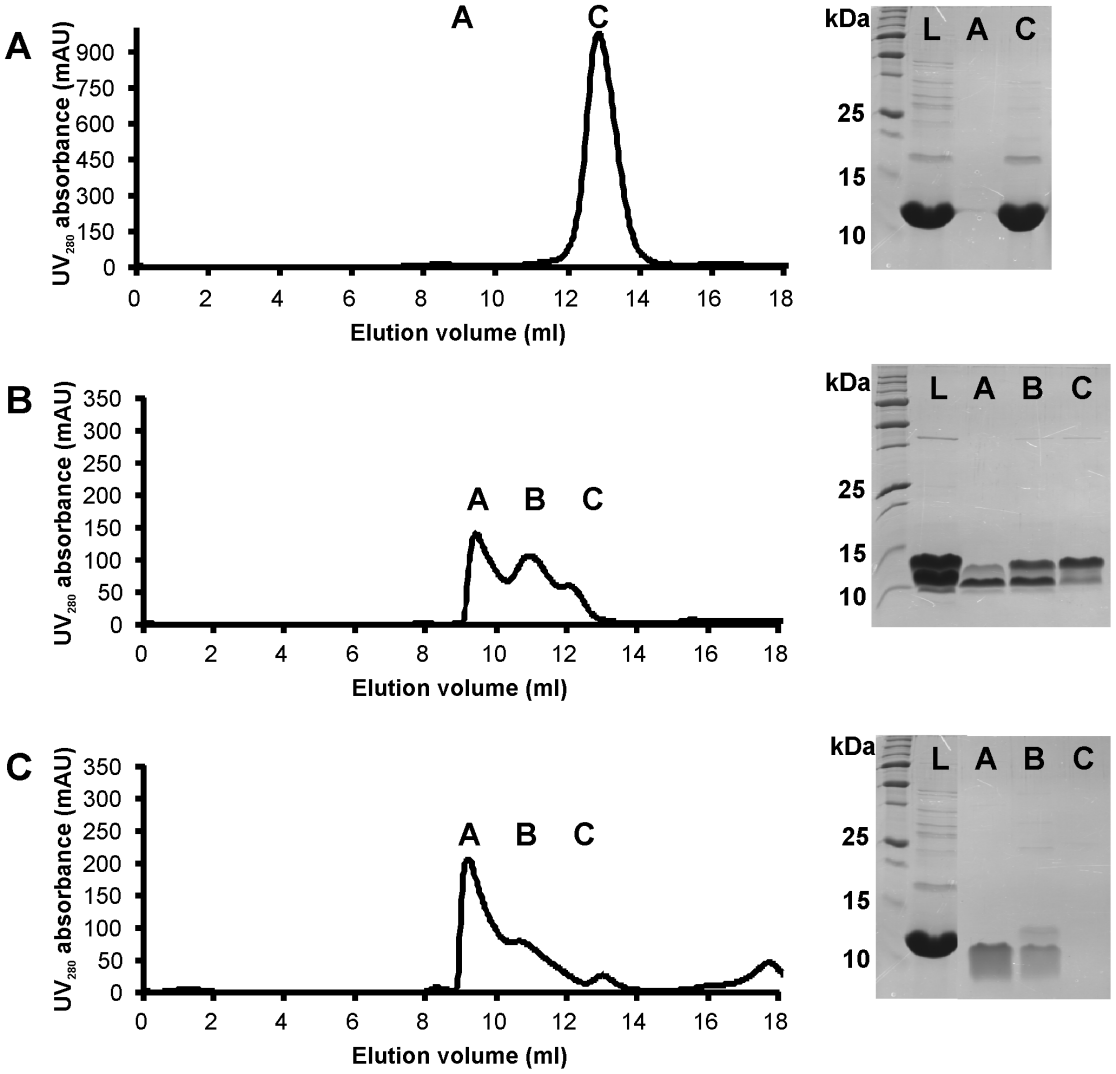
Addition of trypsin to N-Lsr2 facilitates oligomerization. (A) Size exclusion chromatogram of freshly purified Lsr2 N-terminal domain showing a single dimer peak; (B) A chromatogram of the same protein construct used for crystallography (now 14 wks old) demonstrating larger oligomeric forms in solution; (C) The addition of trypsin (1∶500) for 5 min at room temperature to a fresh sample of Lsr2 N-terminal domain accelerates the formation of large oligomeric species in solution. SDS-PAGE gels showing relevant fractions from the purification: L  =  protein loaded on the column; A, B and C represent protein from fractions as labeled on the chromatogram. The N-terminal domain runs at a higher M_r_ than expected on SDS-PAGE gels due to the presence of a 6xHis-tag plus a linker.

To demonstrate the oligomerization of N-Lsr2 in solution, we used size-exclusion chromatography. Overexpressed and freshly purified N-Lsr2 elutes with a symmetrical peak at the expected elution volume for a dimer (Figure 4A). When this chromatogram is compared to that for “mature” N-Lsr2 (a protein sample that is several weeks old, Figure 4B) and proteolyzed N-Lsr2 using trypsin (Figure 4C, 1∶500 trypsin:N-Lsr2, 5 mins incubation), it is clear that oligomers of increasing molecular weight are present in solution and that these oligomers are the result of proteolytic processing.

**Figure 5:**
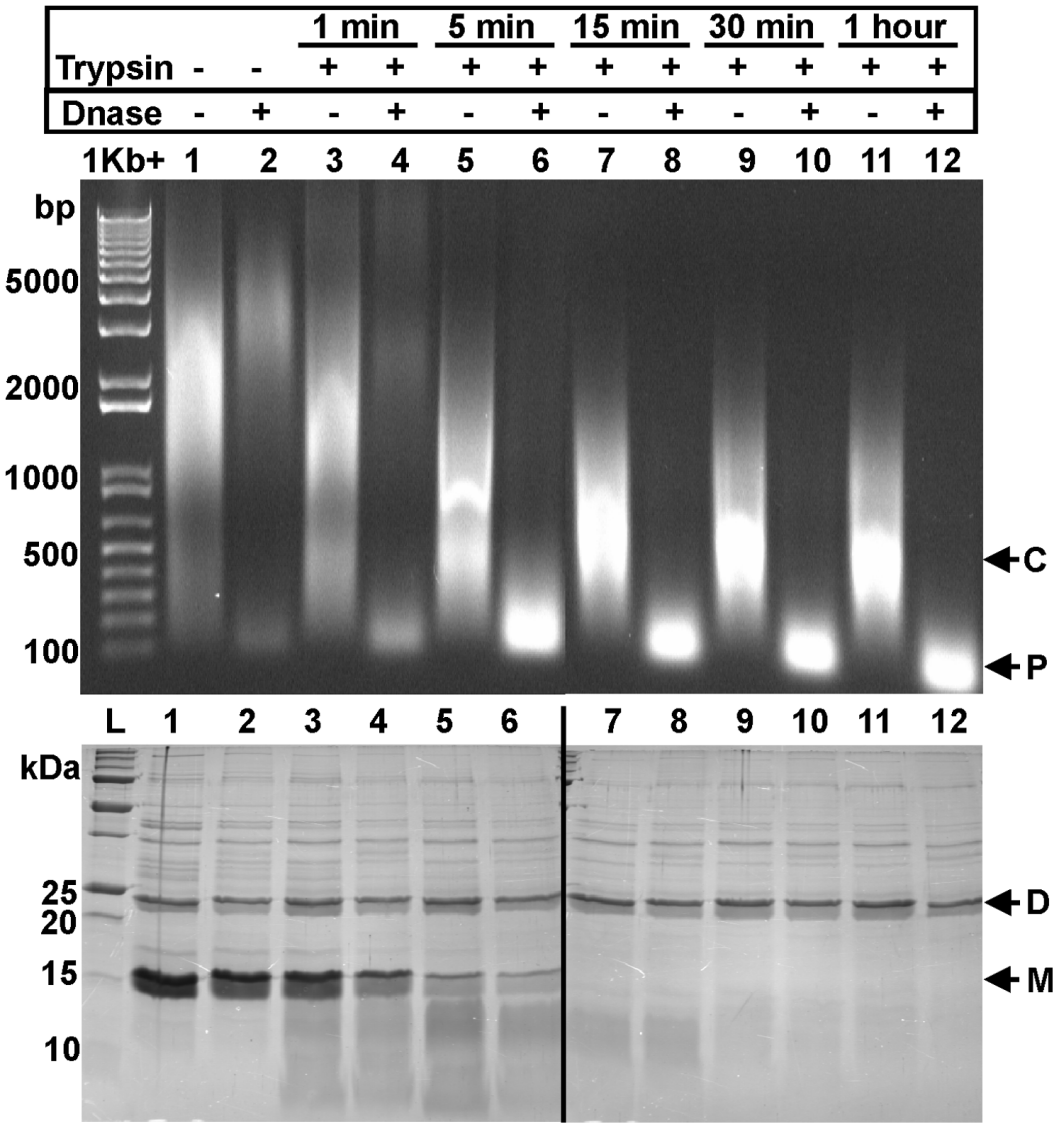
Trypsin digestion of full-length Lsr2 co-purified with *E. coli* genomic DNA. Trypsin (1∶500) initiates the compaction of co-purified DNA by Lsr2 over 1 h as visualised on a 1% agarose gel (SYBR® safe DNA stain). Subsequent digestion of the compacted DNA using DNase shows protection of the DNA by Lsr2. Equivalent samples were run on an SDS-PAGE gel to show the presence of Lsr2 (both monomer and dimer forms) in the sample after trypsin digestion. Components are labeled: C  =  condensation of DNA upon treatment with trypsin; P  =  protection of DNA; D  =  Lsr2 dimer; M  =  Lsr2 monomer).

Next, we sought to characterize the effect of this N-terminal processing and oligomerization on full-length Lsr2 bound to DNA. Full-length Lsr2 that is overexpressed in *E. coli* and purified using Ni^2+^-affinity chromatography, results in a preparation of Lsr2 bound to *E. coli* genomic DNA [20]. If this sample is treated with DNAse for 1 hour, the majority of the DNA is digested and Lsr2 provides little or no protection (Figure 5, lanes 1 & 2). However, proteolytic processing of Lsr2 using trypsin, for increasing periods of time, firstly condenses the DNA and secondly provides increasing levels of protection to DNA towards DNAse degradation (Figure 5, lanes 3–12). Intriguingly, processed Lsr2 appears to protect stretches of DNA of a relatively uniform small size and this may hint at localized zippering of DNA by Lsr2 as opposed to long-range crosslinking of the DNA. SDS-PAGE of equivalent samples shows that prior to proteolytic processing, Lsr2 is labile and runs as a monomer, a dimer and higher oligomers on the gel. However, after processing, Lsr2 runs exclusively as a dimer and higher order oligomers despite the denaturing challenge of SDS and heating prior to running the gel.

**Figure 6:**
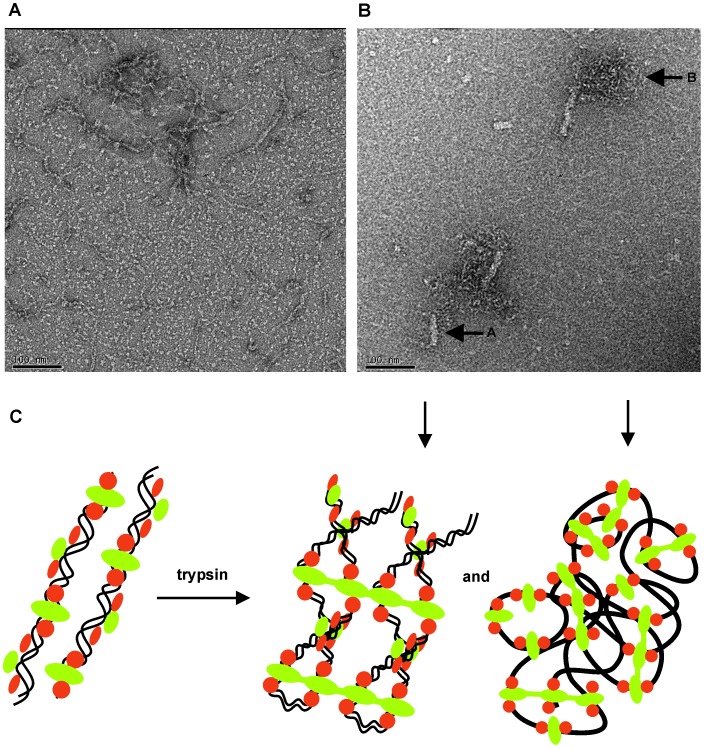
Negatively stained Lsr2/DNA complexes visualized by transmission electron microscopy show large morphological changes upon trypsin treatment. (A) Lsr2 co-purified with *E. coli* genomic DNA; (B) Lsr2 after trypsin digestion (1∶500 ratio) for 30 minutes; Arrows A and B point to condensed structures commonly seen during the trypsin digestion time series. (Scale bars  = 100 nm); (C) A cartoon representation of protein oligomerization and DNA compaction after trypsin digestion. Lsr2 N-terminal dimerization domain is depicted in green and the C-terminal DNA-binding domain is depicted in orange. The downward pointing arrows correlate the features in (B) with their cartoon representations in (C).

The above-mentioned effect of trypsin-induced oligomerization of Lsr2 on DNA topology can be visually confirmed using electron microscopy of heavy-metal stained (negative stain) specimens (Figure 6). Prior to processing, Lsr2-DNA strands are visible with diameters ranging between ∼40 Å and ∼120 Å (diameters estimated from Figure 6A). This dimension is consistent with that for double stranded DNA coated with Lsr2 dimers in the form of single DNA-Lsr2 chains or pairs of DNA-Lsr2 chains lying in close proximity. After proteolytic processing, wider, more regular fibres (∼200 Å diameter) are present along with condensed, knotted bundles of Lsr2-DNA strands (Figure 6B). The diameter of the fibrils suggests higher-order structuring of DNA by Lsr2 oligomers.

The genomic context for *lsr2* in mycobacterial genomes is noteworthy. In all completely sequenced mycobacterial genomes *lsr2* is found adjacent to *clpC*, the ATPase subunit of the Clp protease complex. This suggests a functional connection between these two genes and may point toward the Clp proteases as candidates for post-translational processing of Lsr2. The synteny between *lsr2* and *clpC* is maintained in the Streptomyces species and the Propionibacteria, but not in Rhodococcus species, Actinomyces or Corynebacteria. In the Frankia species, *lsr2* is adjacent to an aminopeptidase of the metallopeptidase M24 family. The homologues for this family in *M. tuberculosis* are PepE (Rv2089c) and PepQ (Rv2535). In addition, several genomes have two copies of *lsr2* (e.g. *Mycobacterium gilvum*, *Mycobacterium smegmatis* and *Streptomyces coelicolor*).

## Discussion

The *lsr2* gene is essential for *Mycobacterium tuberculosis* and is implicated in the regulation of a broad range of cellular processes including cell-wall biosynthesis and the response to antibiotics [20]. Its relationship to histone-like nucleoid-associated proteins from the Enterobacteria also suggests roles in chromosome organization and the regulation of horizontally acquired foreign DNA [23]. Indeed, previous work has shown that Lsr2 from *M. tuberculosis* can crosslink double stranded DNA *in vitro* [7], can physically protect DNA from reactive oxygen species [20] and binds DNA preferentially at AT-rich regions [22], [23]. It has recently been proposed that these functions combine to silence genes acquired by horizontal gene transfer that contain AT-rich tracts of sequence [23]. The fact that Lsr2 is encoded by a number of mycobacteriophages [14] also suggests that these phages may deploy Lsr2 as a gene silencing weapon. Whilst the mode of DNA binding by Lsr2 (via its C-terminal domain) has been well characterized, dimerization and oligomerization via the N-terminal domain are not yet understood. This aspect of Lsr2 biochemistry is particularly important with respect to the mechanism of DNA crosslinking, chromosome organization and gene regulation. In this study, we have revealed atomic details of the interactions that drive Lsr2 dimerization and the mode of self-association of these dimers into linear chains upon limited N-terminal proteolysis which instigates DNA compaction.

We expressed, purified and crystallized the N-terminal domain of Lsr2. Crystals grew only after many weeks and at that time, required an unknown “maturation” process for crystallization to proceed. Nevertheless, these crystals provided high quality X-ray diffraction data that were suitable for structure determination via newly developed *ab initio* phasing techniques [25], [26]. The structure is a dimer that has no homologues in the structural databases and is unlike the oligomerization domain of H-NS from Enterobacteria despite its previously identified functional similarities. The structure provides a rationalization for the sequence conservation seen for this domain – the majority of the conserved residues form tertiary interactions in the structure.

The structure showed linear chains of dimers of the N-terminal domain elaborated in the crystal packing. The chain “links” are constructed from tight association of a ß-strand from each of the N-termini of neighboring dimers. These links can only be readily formed when the N-terminus of the domain is truncated by three residues, and this provided a rationale for the “maturation” required for crystallization. It also presented a possible mechanism for protease-driven oligomerization of Lsr2. We were able to artificially promote oligomerization using trypsin and a second crystallographic structure was determined in a hexagonal space group. This also showed linear chains of N-Lsr2 oligomers identical to those seen in the monoclinic crystals. The difference between the two crystal forms rests with the arrangement of the linear chains in the crystal.

The observation that N-Lsr2 forms higher-order assemblies in solution, particularly upon limited proteolysis, is reflected in the packing of the N-terminal dimerization domain in the crystal structures. The purified full-length protein oligomerises on treatment with trypsin. This oligomerization increasingly protects DNA from DNAse degradation. Further, we observe that DNA compaction and crosslinking can be induced by limited trypsin digestion as directly visualized using electron microscopy. The compaction and protection of DNA as visualized on an agarose gel (Figure 5) raises some intriguing questions. For example, why does Lsr2 only protect relatively small fragments of DNA giving rise to the size difference in Lsr2-DNA complexes before and after DNase treatment (as seen in Figure 5)? We are currently working towards a high-resolution structure of the Lsr2-DNA complex to answer questions such as this.

**Figure 7:**
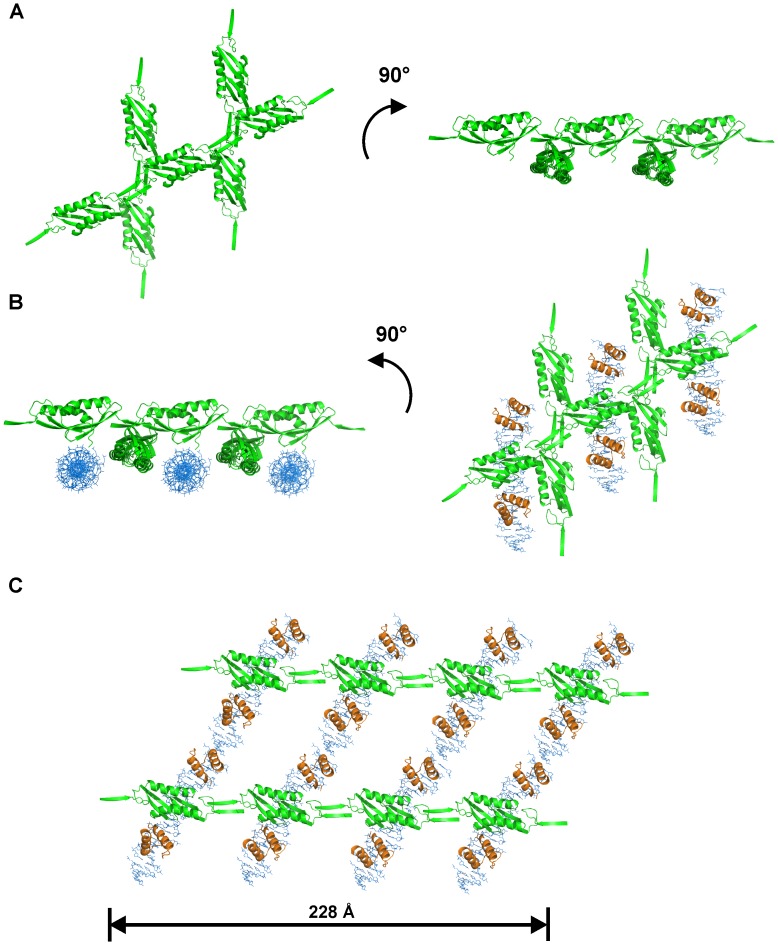
A model for chromosomal DNA organization suggested by hexagonal crystal packing. (A) The N-terminal Lsr2 dimerization domain in the *P*3_1_21 space group in two orientations; (B) Proposed orientation of DNA (in blue) between N-terminal chains and a 90° rotation illustrating this from above. The C-terminal DNA-binding domains (in orange) have been positioned according to Gordon *et al.* [22]; (C) Our model of full-length Lsr2 binding to DNA and forming chains cross-linking multiple strands of DNA. The dimension at the bottom of the figure is consistent with the compact fibrils seen in Figure 6B.

Guided by the electron microscopy images, we have used the hexagonal crystal lattice for N-Lsr2 to speculatively build a model for DNA organization by full-length Lsr2. The hexagonal crystals have a higher solvent content (70.0%) when compared to the monoclinic crystals (38.7%) and there are pores running through the crystal lattice. Intriguingly, these pores are of appropriate dimensions to accommodate long chains of double stranded DNA beneath the N-Lsr2 molecules as well as the Lsr2 C-terminal DNA-binding domains adjacent to the N-Lsr2 molecules. The proposed model (Figure 7), based on these observations, is consistent with the dimensions of the fibers that we observe in electron micrographs (Figure 6). In addition, the model agrees with the mode of DNA binding predicted by Liu and colleagues [22], [23]. Further experiments are ongoing in our laboratory to test the validity of this model.

Under conditions of oxidative stress, starvation or antibiotic challenge, it is advantageous for an organism to physically protect chromosomal DNA and regulate arrays of genes in a coordinated fashion. Lsr2 has been implicated in responses to these stress factors and has been shown to decorate and physically protect DNA from damage. The structure of the oligomerization domain of Lsr2 provides atomic-level details of the dimerization of this domain along with a mechanism for further self-association of these dimers. We observe that formation of higher-order linear structures of N-Lsr2 is facilitated by proteolytic processing of the N-terminus. Also, both protein oligomerization and DNA compaction can be induced by treating full-length Lsr2 with low concentrations of the protease trypsin. There are many proteases encoded in the genome of *M. tuberculosis* that are implicated in the stress response (e.g. Clp, HtrA and FtsH, [29]) and our structure presents a possible mechanism for induced changes in DNA topology and organization (along with accompanying gene regulation) via proteolytic processing of Lsr2 in response to stress.

## Materials and Methods

### Bacterial Strains and Culture Conditions

*E. coli* DH5α was used as a host for all plasmid constructs and *E. coli* BL21 (DE3) was the host for protein expression. *E. coli* was cultured at 30°C or 37°C with shaking (200 rpm) in Luria broth with the addition of amplicillian (100 µg/ml) or kanamycin (50 µg/ml) where appropriate.

### Expression and Purification of Full-length Lsr2

The *M. tuberculosis lsr2* gene (Rv3597c) was amplified by PCR from *M. tuberculosis* H37Rv genomic DNA and cloned into pET30b (Novagen) using the NdeI/XhoI restriction sites, positioning a 6-histidine tag at the C-terminus of the protein [20]. The *lsr2*-pET30b plasmid was transformed into *E. coli* BL21 (DE3) cells and cultured at 37 °C with shaking (200 rpm) in Luria broth with kanamycin (50 µg/ml). A 1 L culture was grown at 37°C until it reached an OD_600_ of 0.4–0.6, cooled to 30°C and induced with 0.5 mM IPTG and grown for 20–22 h. Cells were pelleted by centrifugation and resuspended in 40 mL of 50 mM phosphate buffer (pH 7.4), 200 mM NaCl, 20 mM imidazole buffer. A single EDTA-free protease inhibitor mixture tablet (Roche) was added before cell disruption on ice using sonication. Cellular debris was removed with centrifugation and the supernatant filtered through 1.2 µm and 0.45 µm cellulose acetate filters before purification by Ni^2+^ IMAC using a 5 mL HiTrap chelating HP column (GE Healthcare) and an imidazole gradient. Eluted fractions were examined on 1% agarose and 15% SDS-PAGE gels.

### Expression and Purification of N-terminal Domain of Lsr2

The N-terminal dimerization domain (residues 1–61) of the *M. tuberculosis lsr2* gene (Rv3597c) was amplified by PCR from an *lsr2*-pET30b plasmid template and cloned into pPROExHtb (Invitrogen) using the NcoI/XhoI restriction sites, positioning a 6-histidine tag and rTEV cleavage site at the N-terminus of the protein. The *Nterm2*-pPROExHtb plasmid was transformed into *E. coli* BL21 (DE3) cells and expressed in LB media at 37 °C for 3 hours, and purified as described above for full-length Lsr2. Eluted fractions were examined on 16.5% SDS-PAGE gels. In addition, fractions were pooled from the appropriate IMAC peaks and were concentrated using an ultracentrifugal concentrator (5000 MWCO, polyethersulfone, GE Healthcare) and further purified by size exclusion chromatography (S75 16/60GL or 10/300GL, GE Healthcare) in a 20 mM tris-HCl pH 7.5, 150 mM NaCl buffer. Ultracentrifugation was used to concentrate protein samples for crystallography trials.

### Crystallization of N-terminal Domain of Lsr2

Initial protein crystallization trials (Hampton Research) employed sitting-drop vapor diffusion in 96-well plates and were performed at 18°C. Crystals grew after 4 days by mixing 100 nl of protein solution (15 mg/ml, 20 mM tris-HCl pH 7.5, 150 mM NaCl) with 100 nl of precipitant solution (Hampton screens HR2-086, HR2-130, HR2-134, HR2-136) and equilibrating beside a 100 µl reservoir of precipitant solution. Crystallization fine-screens employed hanging-drop vapor diffusion in 24-well plates at 18°C. Crystals of N-Lsr2 grew after 4 days by mixing 1 µl of >10-week old protein solution (approximately 15 mg/ml, 20 mM tris-HCl pH 7.5, 150 mM NaCl) with 1 µl of precipitant solution (100 mM tris-HCl pH 8.5, 26% PEG 400, 120 mM (NH_4_)_2_SO_4_) and equilibrating over a 500 µl reservoir of precipitant solution.

Co-crystallization trials with N-Lsr2 and trypsin (1 mg/ml, Sigma) at ratios of 1∶500, 1∶5000 and 1∶50000 µg of trypsin to N-Lsr2 were undertaken. Hampton crystallization screens with sitting-drop vapor diffusion in 96-well plates were set up as detailed above and crystals grew after 1 day. Crystallization fine-screens all used trypsin at a ratio of 1∶500 with N-Lsr2 and employed hanging-drop vapor diffusion in 24-well plates at 18 °C. Crystals of N-Lsr2 (co-crystallized with trypsin) grew 6 days after mixing 1 µl of protein solution (15 mg/ml, 20 mM tris-HCl pH 7.5, 150 mM NaCl) with 1 µl of precipitant solution (10 mM CoCl_2_, 100 mM MES pH 6.5, 1.4 M (NH_4_)_2_SO_4_, 7.5% 1-4-dioxane) and equilibrating over a 500 µl reservoir of precipitant solution.

### X-ray Data Collection

N-Lsr2 protein crystals were flash-frozen in liquid nitrogen prior to data collection whereas N-Lsr2 crystals co-crystallized with trypsin were bathed briefly in cryoprotectant (precipitant plus 20% glycerol) prior to data collection. For data collection, crystals were mounted in a stream of cold N_2_ gas at 100 K. Native data from two Nterm2 crystals were collected using a Rigaku Ru 200B rotating anode Cu-Kα radiation source fitted with AXCO PX50 capillary optics and using a Rigaku R-AXIS IIc image plate detector. Two data sets from the initial crystallization conditions (monoclinic crystals) were used to solve the structure via *ab initio* techniques.

A second data set in an alternative space group was collected at the macromolecular crystallography beamline (MX1) at the Australian Synchrotron, Melbourne, Australia, using an ADSC Quantum 210r detector with radiation at a wavelength of 0.9786 Å.

All diffraction data were processed (indexed and integrated) using Mosflm [30] and subsequently scaled using *SCALA* [31] executed in the CCP4 suite of crystallographic software [32]. Data were collected to 1.73 Å and 2.04 Å resolution for rotating anode and synchrotron data respectively, and these resolution limits were determined by the edge of the detectors.

### Structure Solution and Refinement

The monoclinic structure was solved *ab initio* with the program ARCIMBOLDO [26] on the supercomputer Calendula at the FCSCL, León, Spain (www.calendula.fcsc.es). A model alpha helix composed of 14 alanines was used as a search fragment. Location of two fragments led to partial solutions that could be discriminated from the average by a slightly increased correlation coefficient (CC) of the traces obtained by SHELXE [28] but were stuck at mean phase errors of 73° compared to the phases of the final refined and deposited model. Stepwise iterative improvement was attained by interspersing density modification and autotracing with SHELXE jobs with trimming of the partial models obtained to increase their CC until a mean phase error of 42° was reached producing an easily interpretable map.

The structure in a hexagonal space group was determined by molecular replacement using Phenix [33] and the previously determined N-Lsr2 structure as a search model. This was followed by iterative cycles of manual refinement using COOT [34] and Phenix [33].

Coordinate files and structure factors for structures in both monoclinic and hexagonal crystal forms have been deposited in the Protein Data Bank with PDB codes 4E1P and 4E1R respectively.

### Electron Microscopy

Full-length Lsr2 protein samples for negative stain electron microscopy were prepared as follows: 5 µl of sample (2.4 mg/ml) was applied to a 300 mesh copper EM grid covered with carbon-film supported by plastic that had been rendered hydrophilic by glow discharge. After 90 s, the grid was washed with 3 successive 5 µl drops of water applied by pipetting and removing with blotting paper, stained twice with 1.5% uranyl acetate (45 s then 25 s), blotted and air dried. Low-dose images were recorded at a nominal magnification of 42,000 in a FEI TecNai 12 G^2^ microscope equipped with a Lab_6_ gun and operated at 120 kV.

### DNA Protection Assays

Purified full-length Lsr2 protein (2.4 mg/ml) was mixed with trypsin (1 mg/ml, Sigma) at a 1∶500 ratio and incubated at room temperature. Duplicate samples of 15 µl were taken at 1 min, 5 min, 15 min, 30 min and 1 h for 1% agarose and 16.5% SDS-PAGE gel analysis. Samples intended for SDS-PAGE analysis had 5 µl of SDS loading dye added and were heated at 95°C for 5 min. Samples for agarose gel analysis had the trypsin digestion stopped by adding equal amounts of trypsin inhibitor (0.1 mg/ml, Sigma) to the aliquot and were mixed and left at room temperature for a minimum of 15 min before the addition of a gel loading dye. Samples of 30 µl were also taken at 1 min, 5 min, 15 min, 30 min and 1 h for subsequent DNase digestion. All samples for DNase treatment had trypsin inhibitor added at each sampled time point and had a minimum of 45 min treatment time at room temperature before the addition of DNase. To 30 µl samples, 3 µl of 10×DNase buffer and 2 µl of RQ1 DNase (1 U/µl, Promega) was added, mixed and left at room temperature for 90 min. To 15 µl of DNase treated sample, 5 µl of SDS loading dye was added and the sample heated at 95 °C for 5 min. Gel loading dye was added to the remaining 15 µl for agarose gel analysis. Trypsin inhibitor was added to control samples for agarose gel analysis. DNA was visualized in agarose gels using SYBR®safe (Invitrogen) and SDS-PAGE gels were stained with coomassie blue.
